# Adherence to the Mediterranean Diet and its association with sustainable dietary behaviors, sociodemographic factors, and lifestyle: a cross-sectional study in US University students

**DOI:** 10.1186/s12937-024-00962-0

**Published:** 2024-05-27

**Authors:** Cinzia Franchini, Beatrice Biasini, Giovanni Sogari, Rungsaran Wongprawmas, Giulia Andreani, Irina Dolgopolova, Miguel I. Gómez, Jutta Roosen, Davide Menozzi, Cristina Mora, Francesca Scazzina, Alice Rosi

**Affiliations:** 1 Department of Food and Drug, University of Parma, Parma, 43124 Italy; 2 Department of Marketing and Supply Chain Management, School of Business and Economics, Maastricht University, LM Maastricht, 6211 The Netherlands; 3 Charles H. Dyson School of Applied Economics and Management, Cornell University, Ithaca, NY 14853 USA; 4 Marketing and Consumer Research, School of Management, Technical University of Munich, 85354 Freising, Weihenstephan, Germany; 5 Medical School, Building A, Via Volturno 39, Parma, 43125 Italy

**Keywords:** Healthy eating, Sustainable diet, Food behavior, Survey, Young adults, College students

## Abstract

**Background:**

Promoting healthy and sustainable diets is increasingly important and the Mediterranean Diet (MD) has been recognized as an appropriate example that can be adapted to different countries. Considering that the college years are the time when US young adults are most likely to adopt unhealthy eating habits, the present study assessed adherence to the MD and the sustainability of dietary behaviors in a nationally representative sample of US university students, aiming to identify crucial levers for improving their eating behaviors.

**Methods:**

MD adherence and the adoption of healthy and sustainable dietary patterns were assessed through the KIDMED and the Sustainable-HEalthy-Diet (SHED Index questionnaires, respectively, administered through an online survey that also included sociodemographic and behavioral questions. Non-parametric and logistic regression analyses were performed.

**Results:**

A sample of 1485 participants (median (IQR) age 21.0 (19.0–22.0); 59% women) correctly completed the survey. A medium adherence to the MD was the most prevalent (47%). According to multivariate logistic regression analysis, the likelihood of being more compliant with the MD increased when meeting physical activity recommendations, having a high SHED Index score, having the willingness to purchase and eat healthy and sustainable dishes, eating ultra-processed plant-based meat alternatives foods daily, and regularly attending the university canteen.

**Conclusions:**

Encouraging dietary patterns rich in plant-based foods and with a moderate intake of animal products is crucial to increasing the adoption of healthy and sustainable diets, and university dining services represent a suitable setting to build a supportive environment that educates students on human and planetary health.

**Supplementary Information:**

The online version contains supplementary material available at 10.1186/s12937-024-00962-0.

## Introduction

The concept of sustainable diets sheds light on the importance of long-term effects related to the food system [1], which contributes to more than 25% of global greenhouse gas emissions [2] and causes environmental damages, e.g., freshwater eutrophication and soil acidification [3, 4], leading to biodiversity losses. Therefore, the promotion of food patterns that feed the world’s growing population while giving equal priority to human and environmental health is increasingly important [1, 2, 5, 6]. At the same time, the adverse impact of animal-based food on the environment is broadly acknowledged and its substantial reduction is strongly recommended to preserve the well-being of the planet as well as to promote human health [7–10]. In this context, moving to a more plant-based eating model is paramount [6]. The Mediterranean Diet (MD) includes all the key sustainable components (i.e., food security and accessibility, respect for environment and biodiversity, fair trade, locality/seasonality, protection of culture, heritage, and skills) to be recognized as a proper example of a healthy and sustainable diet [7, 11–14]. The MD is mainly characterized by a generous intake of plant-based products, such as grains, fruits, vegetables, legumes, nuts, and herbs, a moderate consumption of white meat, fish, eggs, and dairy products, a low intake of red meat and processed meat, and the use of olive oil as the main seasoning [15, 16]. Furthermore, the MD can contribute to biodiversity protection through the promotion of local, fresh, and seasonal foods [17], and its low environmental impact has been recently recognized [7, 12]. At the same time, high adherence to the MD has been widely associated with a lower risk of non-communicable diseases (NCDs), including diabetes, cancer, cardiovascular and neurodegenerative diseases, and total mortality, in both Mediterranean and non-Mediterranean regions [14, 16, 18–20]. Given this scenario, its adoption should be encouraged in both Mediterranean and non-Mediterranean regions to address dietary-related pathologies and environmental priorities. However, the MD needs to be adapted to different countries according to different cultures, religions, culinary traditions, and food availability and accessibility [21].

In this context, the 2015–2020 Dietary Guidelines for Americans suggested a Healthy Mediterranean-Style Eating Pattern by adapting the Healthy U.S.-Style to the MD principles. More precisely, besides suggesting the proper amounts in which the food groups routinely consumed by Americans should be eaten, increased fruit and seafood consumption and reduced intakes of dairy products are recommended to enhance adherence to the MD [22]. Indeed, contrary to the MD, the Western (American) diet is typically characterized by an overconsumption of refined grains, red and processed meat, processed and ultra-processed foods (UPFs), ready-to-eat meals, snacks, sugar-sweetened beverages, and poor consumption of fruit and vegetables [23–26]. As a result, this diet is a leading cause of the obesity pandemic and the prevalence of NCDs worldwide [24, 27–30]. In particular, for the US, the National Longitudinal Study of Adolescent Health (Add Health) identified a rapid increase in obesity and related diseases (e.g., hypertension and diabetes) during the transition from adolescence to young adulthood in the American population [31]. Starting a work or university career leads to greater independence and autonomy in food choices, which could potentially increase the risk of adopting unhealthy eating habits [32–35]. In addition, the years of college have been identified in the literature as the time when American young adults are most likely to gain weight [36–38]. In this regard, poor nutritional knowledge, lack of time, stress, limited availability and accessibility of healthy foods due to high prices, and easy access to unhealthy junk foods serve as the main barriers to college students’ adoption of healthy eating patterns [37, 39–41].

In light of this, young adulthood should be considered a critical phase for health promotion with lasting effects throughout life [31], and universities should provide a strategic context to support healthy dietary choices and improve college students’ overall well-being [42, 43], also being a benchmark for the entire society and extending benefits beyond the academic community [44]. Based on these considerations, the identification of factors affecting university students’ dietary behaviors allows a better understanding of what is needed to efficiently implement public health strategies addressed to enhance correct dietary habits in younger generations.

To the best of our knowledge, no previous study has explored the eating behaviors of university students living in the United States (US) by enrolling a nationally representative sample. In this context, the present cross-sectional study aimed to address this gap by providing a nationally representative overview of the current food habits of US university students, assessing their level of adherence to a healthy and sustainable dietary pattern such as the MD and its association with sociodemographic and lifestyle-related factors, including the adoption of sustainable dietary behaviors.

## Materials and methods

### Study design and participants

The present study was approved by the local institutional review board (Institutional Review Board for Human Participants, Cornell University, IRB0144167) and conducted according to the ethical principles stated in the Declaration of Helsinki. After receiving the approval, an online self-administered survey was launched on a dedicated platform (Qualtrics software, Version [May 2022] of Qualtrics Copyright © [2022] Qualtrics) and addressed to a representative population of American university students from 18 to 24 years recruited through a marketing agency in May 2022. To obtain a representative sample of the university student population living in the US, gender distribution and the geographic area of residence were considered and a sample size of at least 1400 students was set. Sample representativeness was defined by considering the number of US young adults (*n* = 11,625,381) in the 18–24 year range, as reported in the data record provided by United States Census Bureau (USCB) referred to 1 January 2021. The sample size calculation was performed using G*Power 3.1.9.7 [45], selecting the ANOVA statistical test and the following effect size inputs: f = 0.10; α = 0.05; power (1-β) = 0.8; Df: 3. Before starting the data collection, each participant provided informed consent. Data quality was assessed using attention check questions and recording the time spent filling out the survey. Finally, we used the STROBE-nut reporting guidelines checklist [46] to strengthen data reporting (Supplementary Table S1 - Additional File 1).

### Sociodemographic data, lifestyle variables, and health-related factors

Sociodemographic information was self-reported by each subject. Age, gender identity, state of university location, division of origin, academic status, field of study, living place typology, and financial status were collected as categorical variables, and the number of categories changed depending on the type of information as shown in Table 1. The physical activity level of participants was assessed through the two-item short version of the Nordic Physical Activity Questionnaire (NPAQ-short) [47]. NPAQ-short is a validated tool to assess compliance with World Health Organization (WHO) guidelines on physical activity and sedentary behavior [48], which recommends at least 150 min of Moderate Physical Activity (MPA) or 75 min of Vigorous Physical Activity (VPA) per week, or an equivalent combination of Moderate and Vigorous Physical Activity (MVPA). The closed-ended version of the questionnaire, with five answer options for both questions on time spent on MVPA and VPA in a typical week, was used for the present online survey. Based on the responses, students were classified as compliant with the WHO recommendations if they performed at least 150–300 min of MVPA or 60–90 min of VPA, or a combination of 90–150 min of MVPA and 30–60 min of VPA. Otherwise, the participants’ physical activity was classified as non-compliant with international guidelines.

Additionally, students were asked to report the average weekly frequency of consuming food at the university canteen over the previous 6 months and to express the existence of any physiological (i.e., pregnancy, breastfeeding) and/or pathological (e.g., cardiovascular diseases, diabetes, food intolerances, or allergies) statuses.

### Adherence to the Mediterranean diet

Adherence to the MD was assessed by using the KIDMED questionnaire, a Mediterranean diet quality index validated in children and youths from 2 to 24 years [49]. It consists of 16 yes/no questions based on the dietary principles of the MD. The score assigned to each response is either 1 or -1. Positive scores were assigned to MD-representative eating habits – such as daily consumption of fruit or fresh fruit juice (≥ 1 unit), vegetables (≥ 1 unit), yogurt (2 units), and/or some cheese (40 g), regular weekly consumption of fish (≥ 2 times), nuts (≥ 2 times ) and legumes (> 1 time), having cereals or grains (bread, etc.) and dairy products (yogurt, milk, etc.) for breakfast, habitual consumption of pasta or rice (≥ 5 days per week), and using olive oil as main seasoning at home. On the contrary, a negative score was assigned to eating behaviors not compliant with the MD – such as skipping breakfast or eating commercial baked goods or pastries for breakfast, having a daily and repeated consumption of sweets and candies, and eating at fast-food restaurants more than once a week. The final KIDMED score was calculated and reported as the sum of the scores of each question in a 0-12-point range. In addition, based on the cut-offs defined by the authors [49], respondents were classified into three levels of adherence: low (total score ≤ 3 points), medium (total score 4–7 points), or high (total score ≥ 8 points).

### Nutritional and environmental sustainability of dietary consumption

The sustainability of subjects’ eating behaviors was assessed using a validated questionnaire specifically developed by Tepper and colleagues [50] to measure the adoption of healthy and sustainable dietary patterns, the Sustainable-HEalthy-Diet (SHED) Index. The original version was slightly modified according to local eating habits and food products to better represent the dietary patterns of the involved country population. The SHED Index questionnaire investigates overall dietary consumption and consists of six sections from which six sub-scores were calculated: Healthy Eating (HE), Sustainable Eating (SE), Fruits and Vegetable purchasing location (BFV), Ready meals, Water, and Sodas. The HE and SE scores included 10 and 7 questions, respectively, and focused on the consumption of animal- and plant-based food, attitude towards salt and salty products, low-sugar foods, ultra-processed plant-based meat alternatives foods, organic food, beverages, waste sorting, and local foods. A 4-point Likert scale from “Almost never true” to “Almost always true” was used for both sections. The corresponding scores to the answer options ranged from 0 to 3, except for the scale applied to the first question of the HE sub-section that was reversed. BVF and Water sections consisted of 8 and 4 questions, respectively, investigating the frequency of purchasing fruit and vegetables in different distribution channels (local vs. non-local) and the consumption of different types of water (tap vs. bottled) through a 4-point Likert scale from “Never” (score 0) to “Most of the time” (score 3). Ready meals and Soda scores included 6 and 2 questions, respectively, and aimed at evaluating the frequency of consuming refrigerated, frozen, take-out or home-cooked foods, sugar-sweetened and low-calorie sweetened beverages by applying a 6-point Likert scale from “Never” (score 0) to “Daily or almost daily” (score 5). Specifically, the calculation of the sub-scores for the BFV, ready meals, water, and soda sub-sections required a specific data process according to which the score of each item was multiplied for a specific correction coefficient [50]. The six sub-scores were calculated by summing the obtained scores in each section and the final SHED Index score was computed as the sum of the six sub-scores. Based on this calculation, higher scores were associated with healthier and more sustainable behaviors, and participants were divided into tertiles reflecting low (1st tertile), medium (2nd tertile), and high (3rd tertile) sustainability levels of dietary behaviors. Furthermore, as performed by Tepper and colleagues in two different cohorts [50, 51], respondents were asked to indicate the percentage of plant-based food they include in their usual diet by applying a 0-100% scale. In addition, students needed to report the dietary pattern that most represented them (e.g., omnivore, vegetarian, etc.), and to express their willingness to purchase and consume healthy and sustainable dishes in the following months. The latter question was anticipated by the definitions of sustainable diets and planetary health plates provided by the Food and Agriculture Organization of the United Nations (FAO) [52] and the EAT-Lancet Commission [53]. Lastly, adapting a question from the questionnaire developed by Ohlau and colleagues [54], participants were asked to report their frequency of consumption of ultra-processed plant-based meat alternative products. Specifically, a definition of what is meant by these products was provided in the item, along with examples in line with those most consumed in the US.

### Statistical analysis

Descriptive and inferential statistics were performed. The normality of the data distribution was evaluated and rejected through the Kolmogorov-Smirnov test. On this basis, results were expressed as median and interquartile ranges (IQRs) or as frequency and percentage for continuous and categorical variables, respectively. The non-parametric Kruskal-Wallis H test with Bonferroni *post hoc* test was used to explore and compare differences between continuous variables among subjects with different levels of adherence to the MD (low, medium, and high). The Pearson Chi-square test (χ^2^) was applied to investigate possible associations between the level of adherence to the MD and categorical variables. In addition, the non-parametric Spearman’s rank correlation was applied to assess the degree of association between the continuous variables considered. Finally, based on the significant differences revealed through the analysis mentioned above, univariate and multivariate logistic regression statistics were carried out to investigate which variables increased the likelihood of having a high adherence to the MD. The IBM SPSS Statistics for Macintosh, version 28.0 (Armonk, NY: IBM Corp) was used to perform all statistical analyses, considering a *p*-value less than 0.05 as statistically significant.

## Results

### Participants’ characteristics and adherence to the Mediterranean Diet

A total of 1510 subjects completed the online questionnaire providing all the required information. Of these, 25 records were considered of poor quality as referred to respondents who took less than 40% of the median time or more than 1 h to fill out the questionnaire [55, 56]. After excluding such records, the final sample was composed of 1485 participants representative of university students residing in the US.

The median KIDMED score was 5.0 (IQR: 3.0–7.0) and almost half of the sample (47%) had medium adherence to the MD, while 34% and 20% resulted in having low and high adherence, respectively. Participants’ information such as socio-demographic characteristics, physical activity level, university canteen attendance, and physiological and health conditions are presented in Table 1 for the total samples and by adherence to the MD groups.

**Table 1:** Participants’ characteristics of the entire sample and by the level of adherence to the MD

Variables	**Adherence to the MD** ^**a**^				
	All	Low	Medium	High	*P-*value
	**(n = 1485)**	**(n = 499)**	(n = 695)	(n = 291)	
KIDMED s*core*	5.0 (3.0–7.0)	2.0 (1.0–3.0)^c^	5.0 (4.0–6.0)^b^	9.0 (8.0–9.0)^a^	< 0.001 ^§^
Age (years)	21.0 (19.0–22.0)	21.0 (19.0–22.0) ^c^	21.0 (19.0–22.0)^b^	21.0 (20.0–23.0) ^a^	< 0.001 ^§^
*Gender*					0.001 ^†^
Men	557 (37.5)	153 (30.7)	273 (39.3)	131 (45.0)	
Women	876 (59.0)	321 (64.3)	400 (57.6)	155 (53.3)	
Not-binary/third gender	44 (3.0)	22 (4.4)	18 (2.6)	4 (1.4)	
Prefer not to say	8 (0.5)	3 (0.6)	4 (0.6)	1 (0.3)	
*Geographical area of university location*					0.354 ^†^
Northeast	317 (21.3)	107 (21.4)	145 (20.9)	65 (22.3)	
Midwest	316 (21.3)	105 (21.0)	138 (19.9)	73 (25.1)	
South	598 (40.3)	195 (39.1)	299 (43.0)	104 (35.7)	
West	254 (17.1)	92 (18.4)	113 (16.3)	49 (16.8)	
*Geographical area of origin*					0.310 ^†^
Northeast	308 (20.7)	106 (21.2)	136 (19.6)	66 (22.7)	
Midwest	327 (22.0)	110 (22.0)	145 (20.9)	72 (24.7)	
South	599 (40.3)	192 (38.5)	303 (43.6)	104 (35.7)	
West	251 (16.9)	91 (18.2)	111 (16.0)	49 (16.8)	
*Academic status*					< 0.001 ^†^
Undergraduate student	989 (66.6)	391 (78.4)	431 (62.0)	167 (57.4)	
Graduate student	483 (32.5)	105 (21.0)	256 (36.8)	122 (41.9)	
Other (college students)	13 (0.9)	3 (0.6)	8 (1.2)	2 (0.7)	
*Field of study*					< 0.001 ^†^
Food	211 (14.2)	45 (9.0)	109 (15.7)	57 (19.6)	
Medicine	185 (12.5)	62 (12.4)	87 (12.5)	36 (12.4)	
Scientific-Technological	378 (25.5)	117 (23.4)	182 (26.2)	79 (27.1)	
Human-Social	683 (46.0)	265 (53.1)	304 (43.7)	114 (39.2)	
Other	28 (1.9)	10 (2.0)	13 (1.9)	5 (1.7)	
*Living place typology*					0.023 ^†^
In campus	293 (19.7)	93 (18.6)	131 (18.8)	69 (23.7)	
Outside campus by myself	193 (13.0)	51 (10.2)	99 (14.2)	43 (14.8)	
Outside campus with my partner	135 (9.1)	45 (9.0)	60 (8.6)	30 (10.3)	
Outside campus with my roommates	213 (14.3)	61 (12.2)	112 (16.1)	40 (13.7)	
Parents’ house	611 (41.1)	238 (47.7)	272 (39.1)	101 (34.7)	
Other	40 (2.7)	11 (2.2)	21 (3.0)	8 (2.7)	
*Financial situation*					0.017 ^†^
Not enough to get by	99 (6.7)	45 (9.0)	45 (6.5)	9 (3.1)	
Just enough to get by	495 (33.3)	178 (35.7)	228 (32.8)	89 (30.6)	
Worry about money for fun and extras	610 (41.1)	188 (37.7)	286 (41.2)	136 (46.7)	
Never have to worry about money	221 (14.9)	65 (13.0)	107 (15.4)	49 (16.8)	
I prefer not to answer	60 (4.0)	23 (4.6)	29 (4.2)	8 (2.7)	
*MVPA recommendation*					< 0.001 ^†^
Not met	770 (51.9)	329 (65.9)	343 (49.4)	98 (33.7)	
Met	715 (48.1)	170 (34.1)	352 (50.6)	193 (66.3)	
*Attendance at the university canteen in the last 6 months*					< 0.001 ^†^
Never/rarely	444 (29.9)	207 (41.5)	196 (28.2)	41 (14.1)	
< 1 time/week	215 (14.5)	62 (12.4)	113 (16.3)	40 (13.7)	
1–2 times/week	271 (18.2)	66 (13.2)	148 (21.3)	57 (19.6)	
3–4 times/week	279 (18.8)	80 (16.0)	138 (19.9)	61 (21.0)	
5–6 times/week	132 (8.9)	31 (6.2)	51 (7.3)	50 (17.2)	
Once per day or more	144 (9.7)	53 (10.6)	49 (7.1)	42 (14.4)	
*Pregnancy or breastfeeding*					0.690 ^†^
Yes	26 (1.8)	10 (2.0)	10 (1.4)	6 (2.1)	
No	1459 (98.2)	489 (98.0)	685 (98.6)	285 (97.9)	
*Presence of pathologies, food intolerances or allergies*					0.857 ^†^
Yes	525 (35.4)	178 (35.7)	241 (34.7)	106 (36.4)	
No	960 (64.6)	321 (64.3)	454 (65.3)	185 (63.6)	

Data are presented as the median (IQR) for continuous variables and as number (%) for categorical variables. ^a^ Low total score ≤ 3 points; medium total score 4–7 points; high total score ≥ 8 points. ^§^ Nonparametric Kruskal-Wallis H test for independent sample with Bonferroni post hoc test. Different letters in the same line denote significant differences among adherence to MD groups. ^†^ Person Chi-square test. MVPA: Moderate to Vigorous Physical Activity

The median age of the sample was 21.0 (IQR:19.0–22.0) and most of the respondents were women (59%). Many respondents came from the southern part of the country, both considering the geographical area of origin and university location. Over half of the students (67%) were undergraduates, with the majority attending courses in the human-social disciplinary area (46%), followed by subjects involved in scientific-technological (26%), food science (14%), and medical science (13%) programs.

In addition, more than a third of the participants lived with their parents (41%), and the minority of respondents reported economic insecurity or prefer not to answer (12%). Regarding physical activity, more than half (52%) did not meet MVPA recommendations and declared having diseases or food allergies/intolerances (35%). In addition, 26 women were pregnant or breastfeeding. Lastly, the students’ frequency of attendance at the university canteen was quite variable, with more than half of the participants (56%) attending the canteen at least 1–2 times per week over the previous 6 months.

The results suggest that the level of adherence to the MD was significantly associated with age, gender, academic status, the field of study, compliance with MVPA recommendations, attendance at the university canteen (*p* < 0.001, for all the variables), living place (*p* = 0.023), and financial situation (*p* = 0.017). Students’ characteristics are reported by sex group in Table S2 (Supplementary Table S2 – Additional file 2). Briefly, the results highlighted that, compared with women, men had higher adherence to the MD (*p* < 0.001), were older (*p* = 0.005), and reported a stronger financial situation (*p* = 0.004). A higher percentage of men were graduate students (*p* < 0.001) and attended food-related academic programs (*p* < 0.001) more frequently than females. In addition, the percentage of men living with their parents was lower than women. Men students also met the MVPA recommendations, attended the university cafeteria and ate ultra-processed plant-based meat alternatives more frequently (*p* < 0.001). In addition, based on the SHED index score and sub-scores, men showed more sustainable eating behaviors (*p* = 0.016 for HE score; *p* < 0.001 for SE, BFV, and soda score), whereas water score and ready meals score were higher in the women’s group (*p* < 0.001 for both variables).

**Fig. 1:**
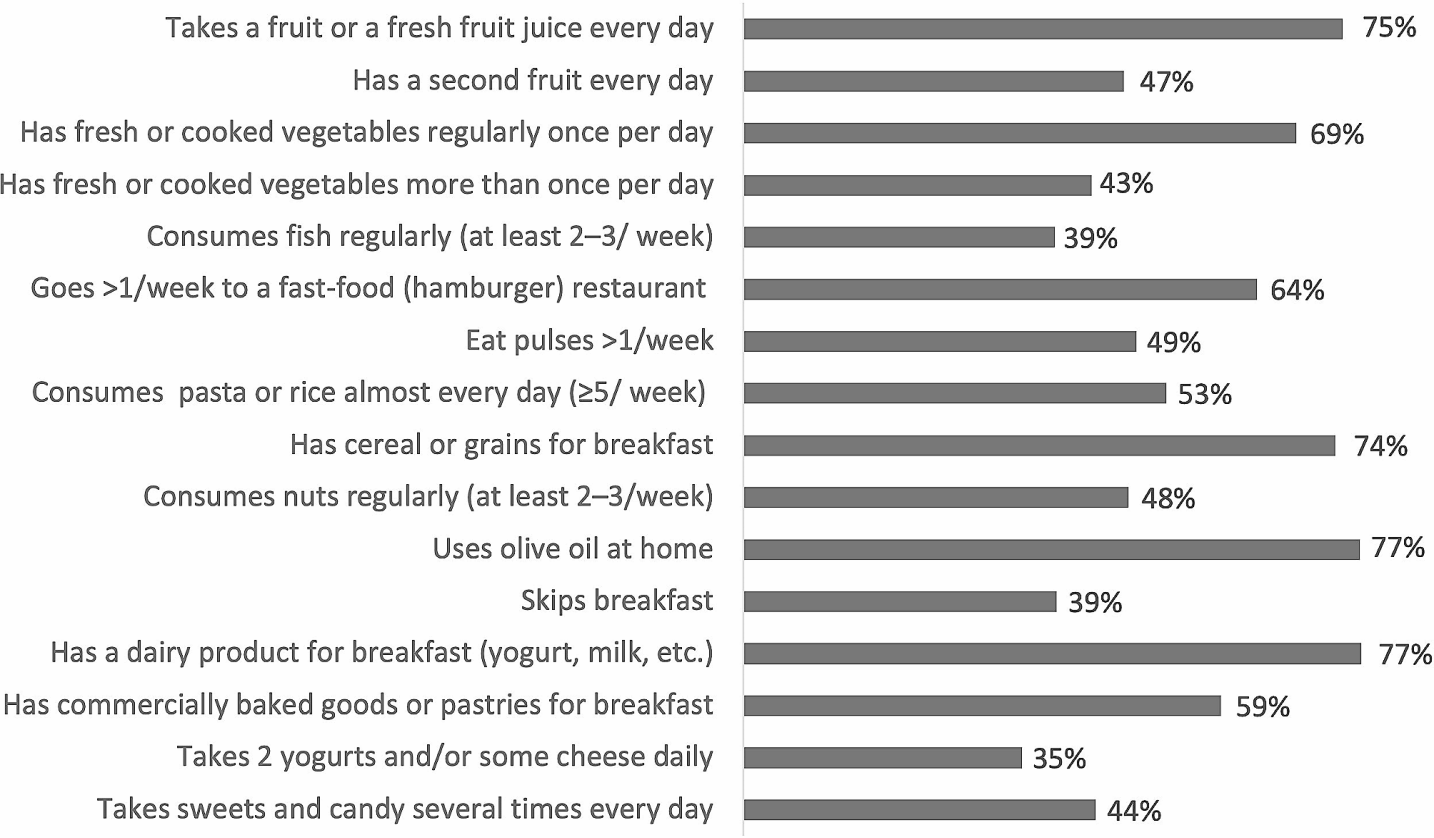
Subjects distribution for each item reported in the KIDMED questionnaire

Responses to each item of the KIDMED questionnaire are reported in Fig. 1.

More than two-thirds of the students consumed one serving of fruits or fresh fruit juice and one serving of vegetables daily, while less than half of the sample reported consuming these foods twice a day. Just over one-third of the participants consumed fish regularly, and about half of the students included legumes and nuts in their diet more than once a week and at least twice a week.

Breakfast was habitually consumed by 61% of the students. More than 70% consumed cereal and dairy products for breakfast, however, only 35% consumed 2 yogurts and/or some cheese every day. Finally, weekly fast-food frequentation and daily repeated consumption of sweets and candies were quite common among US college students (64% and 44%, respectively).

### Nutrition and environmental sustainability of students’ diet

The results presented in Table 2 show the nutritional and environmental sustainability of students’ dietary consumption for the total samples and adherence to the MD groups.

**Table 2:** Dietary behaviors for the entire sample and by the level of adherence to the MD

Variables		Adherence to the MD ^a^			
	**All**	**Low**	**Medium**	**High**	*p-*value
	**(*n*** **= 1485)**	**(n = 499)**	**(n = 695)**	**(n = 291)**	
*SHED index score*	70.0 (55.0–87.0)	58.0 (46.0–71.0) ^c^	71.0 (58.0–85.0) ^b^	91.0 (74.0-110.0) ^a^	< 0.001 ^§^
*SHED sub-scores*					
HE score	15.0 (11.0–19.0)	12.0 (9.0–16.0) ^c^	16.0 (13.0–19.0) ^b^	19.0 (16.0–22.0) ^a^	< 0.001 ^§^
SE score	10.0 (8.0–13.0)	8.0 (6.0–11.0) ^c^	11.0 (8.0–13.0) ^b^	13.0 (11.0–17.0) ^a^	< 0.001 ^§^
BFV score	33.0 (23.5–44.0)	26.0 (19.0–36.0) ^c^	34.0 (24.0–44.0) ^b^	43.0 (33.0–57.0) ^a^	< 0.001 ^§^
Ready meals score	15.0 (10.0–19.0)	13.0 (9.0–18.0) ^c^	15.0 (11.0–19.0) ^b^	17.0 (13.0–21.0) ^a^	< 0.001 ^§^
Water score	6.0 (3.0–9.0)	6.0 (2.0–9.0)	6.0 (3.0–9.0)	6.0 (3.0–9.0)	0.072 ^§^
Soda score	-8.0 (-11.0- -6.0)	-8.0 (-11.0- -5.0) ^b^	-9.0 (-12.0- -6.0) ^a^	-8.0 (-11.0- -4.0) ^c^	< 0.001^§^
*SHED index tertiles* ^*b*^					< 0.001 ^†^
1st tertile	465 (31.3)	261 (52.3)	174 (25.0)	30 (10.3)	
2nd tertile	441 (29.7)	147 (29.5)	236 (34.0)	58 (19.9)	
3rd tertile	579 (39.0)	91 (18.2)	285 (41.0)	203 (69.8)	
% Plant-based foods in the diet	42.0 (27.0–60.0)	31.0 (20.0–46.0) ^c^	45.0 (30.0–61.0) ^b^	59.0 (40.0–75.0) ^a^	< 0.001 ^§^
*Dietary pattern*					< 0.001 ^†^
Omnivore	1138 (76.6)	429 (86.0)	523 (75.3)	186 (63.9)	
Flexitarian	155 (10.4)	27 (5.4)	76 (10.9)	52 (17.9)	
Pescatarian	31 (2.1)	7 (1.4)	15 (1.4)	9 (3.1)	
Vegetarian	76 (5.1)	19 (3.8)	36 (5.2)	21 (7.2)	
Vegan	28 (1.9)	8 (1.6)	13 (1.9)	7 (2.4)	
Raw foodism	5 (0.3)	0 (0.0)	2 (0.3)	3 (1.0)	
Fruitarian	43 (2.9)	8 (1.6)	22 (3.2)	13 (4.5)	
Others	9 (0.6)	1 (0.2)	8 (1.2)	0 (0.0)	
*Willingness to purchase and consume healthy and sustainable dishes*					< 0.001 ^†^
Very unlikely	48 (3.2)	22 (4.4)	16 (2.3)	10 (3.4)	
Unlikely	137 (9.2)	80 (16.0)	51 (7.3)	6 (2.1)	
Undecided	315 (21.2)	156 (31.3)	130 (18.7)	29 (10.0)	
Likely	675 (45.5)	189 (37.9)	369 (53.1)	117 (40.2)	
Very likely	310 (20.9)	52 (10.4)	129 (18.6)	129 (4.3)	
*Frequency of eating ultra-processed plant-based meat alternative foods*					< 0.001 ^†^
Never/Rarely	562 (37.8)	265 (53.1)	237 (34.1)	60 (20.6)	
1–2 times/month	316 (21.3)	103 (20.6)	156 (22.4)	57 (19.6)	
≤ 1 time/week	296 (19.9)	71 (14.2)	154 (22.2)	71 (24.4)	
2–3 times/week	170 (11.4)	37 (7.4)	97 (14.0)	36 (12.4)	
4–5 times/week	89 (6.0)	17 (3.4)	36 (5.2)	36 (12.4)	
Daily or almost daily	52 (3.5)	6 (1.2)	15 (2.2)	31 (10.7)	

Data are presented as the median (IQR) for continuous variables and as number (%) for categorical variables. ^a^ Low total score ≤ 3 points; medium total score 4–7 points; high total score ≥ 8 points. ^b^ 1st tertile ≤ 57; 2nd tertile 58—76; 3rd tertile ≥ 77. ^§^ Nonparametric Kruskal-Wallis H test for independent sample with Bonferroni post hoc test. Different letters in the same line denote significant differences among adherence to MD groups. ^†^ Person Chi-square test. MD: Mediterranean Diet; HE: Healthy Eating; SE: Sustainable Eating; BFV: Fruits and Vegetable purchasing location; SHED: Sustainable-Healthy-Diet

The SHED index and SHED sub-scores, except for the water one, were significantly different among participants grouped according to their level of adherence to the MD (*p* < 0.001), being greater in those having a high adherence. This difference was also confirmed by the significant association observed between the level of adherence to the MD and the distribution among SHED Index score tertiles (*p* < 0.001), and the moderate correlation between KIDMED and SHED Index scores (Spearman’s ρ = 0.506, *p* < 0.001).

The percentage of plant-based foods in the diet was also significantly different among the three MD adherence groups (*p* < 0.001), with the highest percentage being reported by students with high adherence. In terms of other food-related habits, most of the sample was omnivorous (77%), 13% reported following a flexitarian or pescatarian diet, and 7% stated they were vegetarian or vegan. In addition, most of the students indicated that their purchase and consumption of healthy and sustainable dishes in the following months are likely or very likely (61%). In contrast, consumption of ultra-processed plant-based meat alternatives was occasional among university students, with most of them reporting eating such products no more than 1–2 times a month (59%). As for the SHED Index score and sub-scores, Person Chi-square test revealed significant associations between adherence to the MD and the type of dietary pattern followed (e.g., omnivorous, vegetarian, etc.), willingness to purchase healthy and sustainable dishes, and frequency of consumption of ultra-processed plant-based meat alternatives foods (*p* < 0.001).

**Fig. 2:**
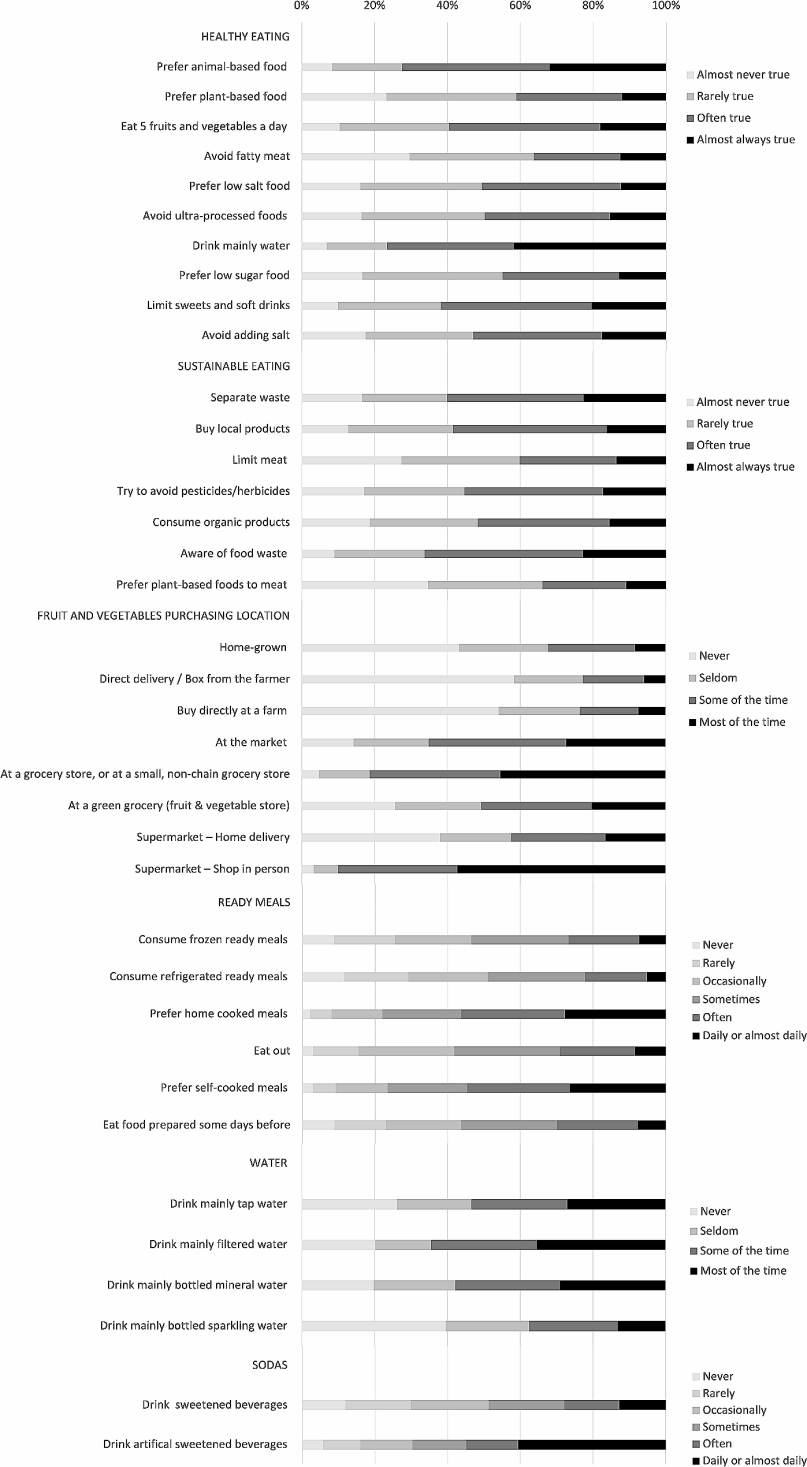
Subjects’ distribution based on the answer option selected for each item reported in the SHED Index questionnaire and grouped by sub-scores

The responses to the SHED Index questionnaire are presented for each sub-score in Fig. 2. As for the healthy and sustainable eating constructs, more than 60% of students reported high frequency (often or almost always true) for the items “eat 5 fruits and vegetables a day,” “drink mainly water”, “limit sweet and soft drinks”, and “prefer animal-based foods”. In contrast, the habit of preferring plant-based products in general and over meat, as well as avoiding meat or fatty meats, was low (almost never or rarely true) in about 60% of the students. In addition, most students (66%) stated that they were aware of food waste and separate waste in most cases (often or almost always true) and reported that they prefer low-pesticide commodities (55%), buying local products (59%), and consuming organic foods (52%) often or almost always.

With respect to fruit and vegetable purchasing locations, the most frequently used channels were supermarkets, grocery stores, or non-chain grocery stores and markets, in descending order. At the same time, most students (68%) reported that they never or seldom grew their own products or bought fruits and vegetables from the farmer directly or through delivery. In terms of the type of meals consumed, more than half of the students preferred home-cooked (56%) or self-cooked meals (55%) in their weekly routine, while in terms of ready-to-eat meals, the distribution of the subjects was quite even among the frequency of consumption. The habit of eating out or eating prepared foods was also rather variable between rarely and often. In addition, students’ beverage preferences included mainly tap (27%), filtered (35%), or bottled mineral (29%) water. Finally, more than 40% of students stated drinking artificially sweetened beverages daily or almost, whereas consumption of sugar-sweetened beverages was less frequent.

### Associations between adherence to the Mediterranean Diet and sustainability of dietary behaviors, sociodemographic, health-related and lifestyle factors

Looking at the results of both univariate and multivariate logistic regression analyses, the strongest predictors of high adherence to the MD were being older, meeting MVPA recommendations, having a higher SHED Index score, consuming mainly plant-based foods, the willingness to purchase and eat healthy and sustainable dishes, eating ultra-processed plant-based meat alternatives daily, and attending the university canteen 5 or more times per week (Table 3). Among other factors, only when assessed separately in the univariate analysis, being woman or non-binary, and living with parents versus staying on campus significantly reduced the likelihood of having high adherence to the MD. On the contrary, according to the univariate analysis, being a graduate student, having financial confidence, and following an exclusively or primarily plant-based diet significantly increased the probability of having a high MD score. Finally, the influence of attending food-related academic programs was found to significantly increase adherence to MD only compared with students enrolled in humanities degree and considering the univariate analysis.

**Table 3:** Logistic regression analysis for being in the high level of adherence to the MD

	Univariate Analysis		Multivariate Analysis	
Variables	OR (95% CI)	*p* Value	OR (95% CI)	*P-*value
Age	1.229 (1.141–1.324)	< 0.001	1.192 (1.086–1.309)	< 0.001
*Gender*				
Men	-1-		-1-	
Women	0.699 (0.538–0.908)	0.007	1.104 (0.796–1.531)	0.555
Not-binary/third gender	0.325 (0.114–0.926)	0.035	0.503 (0.143–1.775)	0.285
*Academic status*				
Undergraduate student	-1-		-1-	
Graduate student	1.663 (1.277–2.166)	< 0.001	1.032 (0.735–1.448)	0.857
Other (college students)	0.895 (0.197–4.075)	0.886	1.588 (0.319–7.901)	0.527
*Field of study*				
Food	-1-		-1-	
Medicine	0.653 (0.406–1.049)	0.078	0.849 (0.519–1.585)	0.575
Scientific-Technological	0.714 (0.482–1.057)	0.092	1.003 (0.626–1.609)	0.989
Human-Social	0.541 (0.376–0.779)	< 0.001	0.697 (0.447–1.088)	0.112
Other	0.587 (0.213–1.619)	0.304	0.584 (0.148–2.305)	0.443
*Living place typology*				
In campus	-1-		-1-	
Outside campus by myself	0.931 (0.603–1.435)	0.745	0.808 (0.473–1.382)	0.437
Outside campus with my partner	0.928 (0.570–1.510)	0.762	0.928 (0.516–1.668)	0.802
Outside campus with my roommates	0.751 (0.485–1.162)	0.198	0.863 (0.510–1.462)	0.584
Parents’ house	0.643 (0.456–0.907)	0.012	1.106 (0.713–1.716)	0.653
*‘Financial situation*				
Not enough to get by	-1-		-1-	
Just enough to get by	2.192 (1.064–4.515)	0.033	1.634 (0.714–3.739)	0.245
Worry about money for fun and extras	2.869 (1.409–5.843)	0.004	2.088 (0.926–4.708)	0.076
Never have to worry about money	2.849 (1.339–6.062)	0.007	1.971 (0.828–4.693)	0.125
*MVPA recommendation*				
Not met	-1-		-1-	
Met	2.535 (1.938–3.317)	< 0.001	1.834 (1.331–2.526)	< 0.001
*Attendance at the university canteen in the last 6 months*				
Never/rarely	-1-		-1-	
< 1 time/week	2.247 (1.404–3.596)	< 0.001	1.664 (0.966–2.867)	0.067
1–2 times/week	2.618 (1.696–4.042)	< 0.001	1.549 (0.932–2.574)	0.091
3–4 times/week	2.750 (1.791–4.223)	< 0.001	1.649 (0.988–2.752)	0.055
5–6 times/week	5.993 (3.722–9.651)	< 0.001	2.855 (1.543–5.281)	< 0.001
Once per day or more	4.047 (2.499–6.554)	< 0.001	2.212 (1.194–4.099)	0.012
*SHED Index score* ^*a*^				
1st tertile	-1-		-1-	
2nd tertile	2.196 (1.384–3.484)	< 0.001	1.241 (0.753–2.044)	0.397
3rd tertile	7.828 (5.209–11.766)	< 0.001	2.923 (1.826–4.678)	< 0.001
% Plant-based foods in the diet	1.029 (1.023–1.035)	< 0.001	1.018 (1.010–1.026)	< 0.001
*Dietary pattern*				
Omnivorous	-1-		-1-	
Plant-based ^b^	2.260 (1.706–2.993)	< 0.001	0.861 (0.596–1.242)	0.422
Others ^c^	1.396 (0.386–5.052)	0.611	1.040 80.196–5.515)	0.963
*Willingness to purchase and consume healthy and sustainable dishes*				
No/maybe	-1-		-1-	
Yes	3.366 (2.399–4.722)	< 0.001	1.827 (1.225–2.725)	0.003
*Frequency of eating plant-based ultra-processed meat alternative foods*				
Never/Rarely	-1-		-1-	
1–2 times/month	1.841 (1.244–2.726)	0.002	1.209 (0.770–1.899)	0.41
≤ 1 time/week	2.640 (1.809–3.853)	< 0.001	1.252 (0.797–1.966)	0.33
2–3 times/week	2.248 (1.426–3.543)	< 0.001	0.738 (0.428–1.274)	0.275
4–5 times/week	5.683 (3.444–9.378)	< 0.001	1.219 (0.645–2.302)	0.542
Daily or almost daily	12.351 (6.676–22.850)	< 0.001	2.725 (1.209–6.145)	0.016

SHED: Sustainable-Healthy-Diet

^a^ 1st tertile ≤ 57; 2nd tertile 58—76; 3rd tertile ≥ 77

^b^ Including vegetarian, vegan, flexitarian, pescetarian, and fruitarian dietary patterns

^c^ Including raw foodism and unspecified dietary patterns

## Discussion

To the best of our knowledge, this is the first study that investigates adherence to the MD and provides insights into food-related behaviors in a representative sample of US university students. Most participants have shown a medium adherence to the MD, whereas concerning predictors of its adoption, meeting physical activity recommendations, having a high SHED Index score, being willing to purchase and consume healthy and sustainable dishes, eating ultra-processed plant-based meat alternatives foods daily, and regularly attending the university canteen were found to be the strongest facilitators to have high adherence to the MD.

Despite the MD has been recognized as a healthy and attainable eating pattern for the American population [22], data on its adoption among US students are limited [57, 58]. In particular, a study conducted in the southeastern US by Bottcher and colleagues [57] explored college students’ dietary habits in terms of adherence to the MD using the Mediterranean Diet Adherence Screener (MEDAS) score [59]. Due to the small sample size and the use of a different MD score, the comparison with our results is difficult. However, in line with our findings, about half of the sample had a medium level of adherence to the MD, while the percentage of students with a low adherence was greater [57].

Looking at the individual dietary habits characterizing the Mediterranean dietary pattern, our findings are consistent with previous research showing that the eating habits of young adults in the US are poor in fruit, vegetables, and legumes [60–63]. As found in previous studies, many students usually go to fast food more than once a week [64, 65]. The updated picture of students’ food habits provided by the present study highlights some negative aspects. Indeed, the percentage of college students skipping breakfast was higher than that of young adults (20–39 years old) enrolled in the prospective National Health and Nutrition Examination Survey (NHANES) 1999–2006 [66]. Conversely, fewer college students regularly consume nuts compared to participants (18–30 years old) in the Coronary Artery Risk Development in Young Adults (CARDIA) [67]. This minor tendency to have breakfast and consume nuts regularly could be related to the younger age of our sample. In this regard, a similar positive association between age and breakfast and nut consumption was previously reported in the literature for the US population [67, 68]. In addition, the low consumption of fish and dairy products found in our survey is in line with the average intake of US young adults reported in the results of the most updated national survey [63].

In this context, the MD can serve as a reference pattern for promoting healthier and more sustainable diets among US university students. Promising results were highlighted by Petroka and colleagues [58], who evaluated the eating habits of a sample of US college students before and after a 3-week exchange period in Italy. This short-term exposure to the MD led to significant dietary changes including lower consumption of meat products and higher intakes of fruits, grains, and olive oil as well as overall greater adherence to the MD.

Regarding predictors of adherence to the MD, consistent with our findings, other studies [69–71] showed that young adults who had a higher MD score were more likely to be physically active. In contrast to our results, previous studies found higher adherence to the MD in women, both in an American study [57] and in other populations in Mediterranean regions [69, 70, 72]. Nevertheless, the association between the adoption of the MD and sex is not univocal, as one study performed in Mediterranean regions [73] revealed that the role of gender was different depending on the score applied. Moreover, our results are consistent with the adherence observed by Bottcher and colleagues, in which the MD score was higher in older students [57]. In addition, possible differences in adherence to the MD depending on the field of study have already been pointed out by previous studies [74, 75]. The evidence suggested that deepening health-nutrition topics increases the likelihood of high adherence to the MD. It should be noted that these studies referred to students in biomedical careers and not food science students, as emphasized by our results. However, in line with our work, Castro-Cuesta and colleagues [74] reported lower adherence in students in humanities programs than in health science students.

Above all, financial constraints increase food insecurity and poorer dietary outcomes such as low intake of fruit, vegetables, and whole grains, and overconsumption of added sugars, sugary drinks, and fast foods [76–79]. As a matter of fact, those US students who stated to have enough money to get by or a better financial status were more adherent to the MD. Similarly, to what was reported in the literature, US students residing on campus had healthier eating habits [80], especially when compared with peers living with their parents [81]. Furthermore, the positive role of on-campus dining services in encouraging users to adopt healthy eating habits has already been reported in the literature [82]. Furthermore, the availability and accessibility of healthy foods are one of the main barriers to adopting a Mediterranean dietary style [83], especially in the US [84]. In this context, several virtuous examples have demonstrated the key role of food services at US universities in ensuring healthy, sustainable, and affordable food for their communities [85].

Similarly to our results, a significant positive correlation between the sustainability of food behaviors, assessed through the SHED Index score, and the MD adherence score was previously observed in two adult populations of the Mediterranean Basin [50, 51]. Furthermore, earlier studies performed on Belgian [86] and Israeli [87] cohorts suggested that the adoption of exclusively (i.e., vegan and fruitarian) or partially (i.e., vegetarian, flexitarian, pescetarian) plant-based diets is a positive predictor of compliance with the MD. This result is not surprising given the large share of plant-based foods associated with these dietary patterns.

Additionally, in line with our findings, a recent systematic review [88] pointed out that university students with healthier lifestyles and diets adopt more sustainable food consumption behaviors.

Lastly, regular consumption of ultra-processed plant-based meat alternative foods emerged as a factor that positively influenced adherence to the MD. Our findings may reflect the increasing penetration of plant-based meat alternatives in the US market [89] as well as consumers’ demands [90]. Notably, a recent publication [90] emphasized that younger consumers and those who report following alternative diets, such as vegan, vegetarian, or flexitarian/semi-vegetarian, are the most likely to consume ultra-processed plant-based protein alternatives.

Although the health benefits associated with large consumption of plant-based food are widely recognized, generalization is not possible [91]. As recently emphasized by WHO [91], it is essential to prefer minimally processed plant-based foods such as whole cereals, fruit and vegetables, pulses, seeds, and nuts, and to limit the consumption of UPFs such as sugary drinks, snacks, and sweets, as well as ultra-processed plant-based foods that mimic animal products. These products are generally energy-dense, lacking in fiber and micronutrients, and high in saturated fatty acids, salt, and added sugars [92] and several previous studies reported a positive association between high consumption of UPFs and increased risk of multimorbidity [93–95]. However, the Nova classification based on which UPFs are defined leads to the inclusion of a very heterogeneous type of products in this group [96]. Bearing this in mind, a recent multinational cohort study [97] attempted to better understand the association between the consumption of UPFs and the incidence of NCDs, reporting results for several food groups. The authors’ findings corroborated the negative health impact of some categories of UPF, such as sugary and artificial beverages, animal-based products, sauces, spreads, and condiments. On the contrary, ultra-processed plant-based alternatives as well as bread and cereals were not associated with an increased risk [97]. In this context, the role of ultra-processed plant-based alternatives within a healthy diet as a possible solution to reduce the consumption of meat products should be taken into account and better explored in future research.

To the authors’ knowledge, no previous studies have assessed adherence to the MD and sustainable food consumption in a large representative sample of US university students. Socio-demographic information and behavioral variables associated with eating habits were also evaluated to provide a better overview of the factors facilitating or discouraging sustainable dietary behaviors in university students. Despite the novelty of our research, some inherent limitations need to be outlined. To begin with, the cross-sectional nature of the study does not allow for a clear causal relationship between a healthy and sustainable diet and possible facilitators of its adoption but rather should be considered as a representative baseline to design future intervention studies. In addition, the applied SHED Index score evaluated the fruits and vegetables purchasing location, rewarding the choice of local products but without considering the seasonality aspect. As a matter of fact, when it comes to sustainable food consumption, the two aspects should be considered together, adopting a more inclusive concept of local seasonality [98]. Also, as pointed out by Alexandropoulos and colleagues, although this score encompasses several aspects of a sustainable diet, it is not based on dietary intake and thus fails to quantify the greenhouse gas emissions associated with the consumption. Nevertheless, as pointed out by the same authors, all currently available indices for assessing diet sustainability have some limitations and a gold standard is still lacking. Given the study design, the SHED index score was considered the most appropriate. However, further implementation of the questionnaire to address the previously highlighted shortcomings and validate it on other sample populations would be appropriate. Despite this, the positive correlations found between the MD and SHED Index scores, as already reported in the literature [50, 51], are promising. On the other hand, the use of the validated KIDMED questionnaire specifically developed and widely used to assess adherence to the MD (primary outcome) in young populations, is a strength of the project. However, given the wide age range to which it can be applied (2–24 years old), the questionnaire does not investigate alcohol consumption, information particularly relevant to the young adult population. Furthermore, the first item of the questionnaire is unable to distinguish between fresh fruit and fresh fruit juice. While for the former plenty of consumption is recommended by worldwide food and health organizations [99], for the latter a recent meta-analysis suggests reducing consumption to limit excessive calorie intake and prevent weight gain [100]. From this perspective, it is desirable to develop a more detailed tool able to discriminate the two types of products. Also, the inverse association between bread and ultra-processed grains and the risk of multimorbidity evidenced in the multinational cohort study mentioned above [97] may provide food for thought for reconsidering the item on commercially baked goods or pastries in the total score.

In addition, the KIDMED questionnaire is limited to certain food categories and does not allow the collection of quantitative food data, that could have been useful for assessing compliance with dietary guidelines and national nutritional recommendations besides adherence to the MD. However, taking into account the purpose of the study and the amount of information required from participants, the use of KIDMED was considered the best option. Lastly, the application of a self-administered online survey represents a convenient solution being an easy-to-use tool that requires little effort, but it may increase the possibility of recall and misreporting bias.

## Conclusion

The current study assessed adherence to the MD in a representative sample of US university students and investigated the relationship between the MD score and sustainability of food behaviors, as well as other factors acting as predictors of students’ eating habits.

Overall, a medium adherence to the MD and a strong relationship between adherence to the MD and sustainable dietary behaviors were observed. These results confirmed that the MD is a suitable eating pattern to consider when implementing public interventions to shift university students’ eating habits toward healthy and sustainable diets. In this connection, a major promotion of the MD as a sustainable dietary pattern may be an effective strategy for its revitalization, especially among young adults such as university students, who show greater consciousness and attention to current environmental issues than older populations. Considering the positive influence that regular university canteen attendance has on students’ eating habits, university dining services represent a unique opportunity to build a supportive environment to educate students on the effects of their actions and foster human and planetary health.

### Electronic supplementary material

Below is the link to the electronic supplementary material.

**Additional file 1**: **Supplementary Table S1**. STROBE-nut checklist.

**Additional file 2**: **Supplementary Table S2**. Participants’ characteristics reported for men and women.

## Data Availability

The data collection tools used, and dataset generated during the present study may be made available by the corresponding author on reasonable request.

## Abbreviations

MD: Mediterranean Diet
NCDs: Non-communicable diseases
UPFs: Ultra-processed foods
US: United States
USCB: United States Census Bureau
NPAQ: Nordic Physical Activity Questionnaire
WHO: World Health Organization
MPA: Moderate Physical Activity
VPA: Vigorous Physical Activity
MVPA: Moderate and Vigorous Physical Activity
SHED: Sustainable-HEalthy-Diet
HE: Healthy Eating
SE: Sustainable Eating
BFV: Fruits and Vegetable purchasing location
FAO: Food and Agriculture Organization of the United Nations
IQR: Interquartile Range
MEDAS: Mediterranean Diet Adherence Screener
NHANES: National Health and Nutrition Examination Survey
CARDIA: Coronary Artery Risk Development in Young Adults
