# Supplementary material for: Adherence to the Mediterranean Diet and its association with sustainable dietary behaviors, sociodemographic factors, and lifestyle: a cross-sectional study in US University students
Source: Nutr J. 2024 May 27;23:56. doi: 10.1186/s12937-024-00962-0 (PMC11129378; doi:10.1186/s12937-024-00962-0)
Supplement: Supplementary file 2 — Additional file 2: Supplementary Table S2. Participants’ characteristics reported for men and women. [file 12937_2024_962_MOESM2_ESM.docx]

**Supplementary Table S2**

**Table S2** Participants’ characteristics reported for men and women.

| **Variables** | **All**  **(n = 1433)** | **Men**  **(n = 557)** | **Women**  **(n = 876)** | *p-V*alue |
| --- | --- | --- | --- | --- |
| KIDMED s*core* | 5.0 (3.0-7.0) | 5.0 (3.0-7.0) | 5.0 (2.0-7.0) | <0.001 ^§^ |
| *Level of adherence to MD ^a^*  Low  Medium  High | 474 (33.1)  673 (47.0)  286 (20.0) | 153 (27.5)  273 (49.0)  131 (23.5) | 321 (36.6)  400 (45.7)  155 (17.7) | <0.001 ^†^ |
| Age (years) | 21.0 (20.0-22.0) | 21.0 (20.0-22.0) | 21.0 (19.0-22.0) | 0.005 ^§^ |
| *Geographical area of university location*  Northeast  Midwest  South  West | 311 (21.7)  298 (20.8)  581 (40.5)  243 (17.0) | 115 (20.6)  116 (20.8)  233 (41.8)  93 (16.7) | 196 (22.4)  182 (20.8)  348 (39.7)  150 (17.1) | 0.831 ^†^ |
| *Geographical area of origin*  Northeast  Midwest  South  West | 302 (21.1)  310 (21.6)  580 (40.5)  241 (16.8) | 105 (18.9)  116 (20.8)  244 (43.8)  92 (16.5) | 197 (22.5)  194 (22.1)  336 (38.4)  149 (17.0) | 0.175 ^†^ |
| *Academic status*  Undergraduate student  Graduate student  Other (college students) | 951 (66.4)  469 (32.7)  13 (0.9) | 319 (57.3)  234 (42.0)  4 (0.7) | 632 (72.1)  235 (26.8)  9 (1.0) | <0.001 ^†^ |
| *Field of study*  Food  Medicine  Scientific-Technological  Human-Social  Other | 208 (14.5)  181 (12.6)  362 (25.3)  655 (45.7)  27 (1.9) | 134 (24.1)  35 (6.3)  149 (26.8)  228 (40.9)  11 (2.0) | 74 (8.4)  146 (16.7)  213 (24.3)  427 (48.7)  16 (1.8) | <0.001 ^†^ |
| *Living place typology*  In campus  Outside campus by myself  Outside campus with my partner  Outside campus with my roommates  Parents’ house  Other | 280 (19.5)  190 (13.3)  133 (9.3)  204 (14.2)  588 (41.0)  38 (2.7) | 131 (23.5)  96 (17.2)  41 (7.4)  73 (13.1)  202 (36.3)  14 (2.5) | 149 (17.0)  94 (10.7)  92 (10.5)  131 (15.0)  386 (44.1)  24 (2.7) | <0.001 ^†^ |
| *Financial situation*  Not enough to get by  Just enough to get by  Worry about money for fun and extras  Never have to worry about money  I prefer not to answer | 55 (3.8)  93 (6.5)  477 (33.3)  591 (41.2)  217 (15.1) | 21 (3.8)  25 (4.5)  165 (29.6)  247 (44.3)  99 (17.8) | 34 (3.9)  68 (7.8)  312 (35.6)  344 (39.3)  118 (13.5) | 0.004 ^†^ |
| *MVPA recommendation*  Not met  Met | 735 (51.3)  698 (48.7) | 215 (38.6)  342 (61.4) | 520 (59.4)  356 (40.6) | <0.001 ^†^ |
| *Attendance at the university canteen in the last 6 months*  Never/rarely  < 1 time/week  1-2 times/week  3-4 times/week  5-6 times/week  Once per day or more | 423 (29.5)  208 (14.5)  262 (18.3)  271 (18.9)  130 (9.1)  139 (9.7) | 126 (22.6)  69 (12.4)  101 (18.1)  118 (21.2)  73 (13.1)  70 (12.6) | 297 (33.9)  139 (15.9)  161 (18.4)  153 (17.5)  57 (6.5)  69 (7.9) | <0.001 ^†^ |
| *Presence of pathologies. food intolerances or allergies*  Yes  No | 933 (65.1)  500 (34.9) | 420 (75.4)  137 (24.6) | 513 (58.6)  363 (41.4) | <0.001 ^†^ |
| *SHED index score* | 70.0 (55.0-87.0) | 72.0 (57.0-92.0) | 68.0 (53.0-83.0) | <0.001 ^§^ |
| *SHED sub-scores*  HE score  SE score  BFV score  Ready meals score  Water score  Soda score | 15.0 (11.0-19.0)  10.0 (8.0-13.5)  24.0 (33.0-44.0)  15.0 (10.0-19.0)  6.0 (3.0-9.0)  -8.0 (-11.0- -5.0) | 16.0 (12.0-19.0)  11.0 (8.0-14.0)  36.0 (25.5-48.0)  14.0 (10.0-18.0)  6.0 (3.0-8.0)  -8.0 (-11.0- -5.0) | 15.0 (11.0-18.0)  10.0 (7.0-13.0)  22.0 (31.0-42.0)  15.0 (11.0-20.0)  6.0 (3.0-9.0)  -9.0 (-11.0- -6.0) | 0.016 ^§^  <0.001^§^  <0.001^§^  <0.001 ^§^  0.575 ^§^  <0.001 ^§^ |
| *SHED index tertiles ^b^*  1^st^ tertile  2^nd^ tertile  3^rd^ tertile | 447 (31.2)  425 (29.7)  561 (39.1) | 151 (27.1)  163 (29.3)  243 (43.6) | 296 (33.8)  262 (29.9)  318 (36.3) | 0.008 ^†^ |
| % Plant-based foods in the diet | 42.0 (27.0-60.0) | 41 (26.0-64.0) | 42 (27.0-60.0) | 0.559 ^§^ |
| *Dietary pattern*  Omnivore  Flexitarian  Pescatarian  Vegetarian  Vegan  Raw foodism  Fruitarian  Others | 1099 (76.7)  75 (5.2)  26 (1.8)  149 (10.4)  28 (2.0)  5 (0.3)  42 (2.9)  9 (0.6) | 436 (78.3)  30 (5.4)  12 (2.2)  54 (9.7)  6 (1.1)  0 (0.0)  16 (2.9)  3 (0.5) | 663 (75.7)  45 (5.1)  14 (1.6)  95 (10.8)  22 (2.5)  5 (0.6)  26 (3.0)  6 (0.7) | 0.312 ^†^ |
| *Willingness to purchase and consume healthy and sustainable dishes*  Very unlikely  Unlikely  Undecided  Likely  Very likely | 47 (3.3)  133 (9.3)  304 (21.2)  649 (45.3)  300 (20.9) | 18 (3.2)  62 (11.1)  103 (18.5)  244 (43.8)  130 (23.3) | 29 (3.3)  71 (8.1)  201 (22.9)  405 (46.2)  170 (19.4) | 0.050 ^†^ |
| *Frequency of eating ultra-processed plant-based meat alternatives foods*  Never/Rarely  1-2 times/month  ≤ 1 time/week  2-3 times/week  4-5 times/week  Daily or almost daily | 541 (37.8)  304 (21.2)  287 (20.0)  165 (11.5)  87 (6.1)  49 (3.4) | 175 (31.4)  116 (20.8)  127 (22.8)  70 (12.6)  42 (7.5)  27 (4.8) | 366 (41.8)  188 (21.5)  160 (18.3)  95 (10.8)  45 (5.1)  22 (2.5) | <0.001 ^†^ |

Data are presented as the median (IQR) for continuous variables and as number (%) for categorical variables. ^a^ Low total score ≤ 3 points; medium total score 4–7 points; high total score ≥ 8 points. ^b^ 1st tertile ≤ 55; 2nd tertile 55—73; 3rd tertile > 73. ^§^ Nonparametric Kruskal-Wallis H test for independent sample with Bonferroni post hoc test. Different letters in the same line denote significant differences.
